# Adaptions in eye-movement behavior during face-to-face communication in noise

**DOI:** 10.3389/fpsyg.2025.1584937

**Published:** 2025-10-03

**Authors:** Valeska Slomianka, Tobias May, Torsten Dau

**Affiliations:** Hearing Systems Section, Department of Health Technology, Technical University of Denmark, Kongens Lyngby, Denmark

**Keywords:** eye-tracking, turn-taking, communication difficulty, face-to-face communication, triadic conversations

## Abstract

In face-to-face conversations, gaze serves a dual role: it conveys non-verbal messages and facilitates the perception of visual cues that support speech comprehension and smooth turn-taking. Typically, listeners direct their gaze toward the current talker to signal interest in taking the next turn, while talkers monitor listeners for signs of engagement. However, how gaze behavior and its coordination with turn-taking adapt to challenging acoustic environments remains poorly understood. In this study, ten groups of three young, normal-hearing Danish participants engaged in six discussions on several topics, each lasting approximately 7 min. Participants’ eye movements were recorded using Tobii Pro Glasses 3 wearable eye-tracking devices. Conversation difficulty was manipulated by introducing two levels of eight-talker background noise (‘8-talker babble’). Each group participated in three conversations in noise and three in quiet. The analysis revealed that in noisy conditions, participants looked more frequently at their conversational partners and made more eye movements overall. Gaze behavior also became more tightly synchronized with turn-taking: participants showed reduced gaze avoidance at the beginning of their own turns, and both talkers and listeners increasingly oriented their gaze towards the next talker at the end of a turn. These findings indicate that background noise significantly shapes gaze behavior, suggesting an increased reliance on visual information to manage conversational dynamics and comprehend speech. This highlights the critical role of gaze in communication and its potential to inform the design of communication aids and strategies, especially for individuals with communication challenges in noisy environments.

## Introduction

Every day, we engage in conversations without fully appreciating the complexity our perceptual system must navigate. Face-to-face communication is a rich, multimodal interaction in which speech is integrated with non-verbal behaviors such as gaze, gestures, and facial expressions (Holler and Levinson, 2019; Kendrick et al., 2023; Nota et al., 2021; Trujillo et al., 2020). Rather than overloading our cognitive system, these multimodal cues help facilitate conversational flow and support speech comprehension, with multimodal messages typically processed faster than unimodal ones. In fluent conversations, interlocutors alternate rapidly between listening and talking, with gaps between turns lasting only about 250 ms (Aubanel et al., 2011; Petersen et al., 2022; Stivers et al., 2009). This requires interlocutors to process speech and non-verbal signals while simultaneously planning responses and coordinating turn-taking. Challenges such as background noise or hearing impairment can disrupt this coordination, leading individuals to rely more on visual cues (Hadley et al., 2019), slow down turn-taking (Sørensen et al., 2021), and experience more frequent conversational breakdowns (McKellin et al., 2007; Miles et al., 2023). These disruptions can increase stress and fatigue and, over time, contribute to social withdrawal and reduced quality of life (Dalton et al., 2003; Jones et al., 1981; Stansfeld and Matheson, 2003). Accurately assessing such communication difficulties is essential for diagnosing and treating communication disorders. However, current clinical tests primarily focus on listening abilities and often fail to predict an individual’s capacity to actively engage in real-life conversations – highlighting the need for objective measures that more comprehensively capture communication challenges (Keidser et al., 2020).

Gaze behavior provides valuable insights into cognitive processes, such as memory, attention, and cognitive load (Ballard et al., 1995; Hayhoe and Ballard, 2005; Mahanama et al., 2022; Šabić et al., 2020). Eye movements play a significant role in structuring visual input and supporting memory encoding. Rather than relying solely on internal memory, individuals often use the visual environment as an “external memory,” repeatedly sampling relevant information to reduce cognitive effort (O’Regan, 1992). This strategy reflects a trade-off between gaze and working memory: people tend to rely on gaze when visual information is easily accessible and shift to internal memory when it is not (Droll and Hayhoe, 2007). The focus of visual attention is closely aligned with auditory attention (Hendrikse et al., 2018, 2019, 2022), and our perceptual systems integrate both into a unified audiovisual percept (Stacey et al., 2020; Stein and Meredith, 1993). When auditory information is degraded, such as in the presence of background noise, individuals rely more heavily on visual cues to support comprehension, which leads to changes in gaze behavior. For example, in noisy conditions, listeners tend to spend more time looking at the talker - especially at the mouth – rather than the eyes (Buchan et al., 2008; Hadley et al., 2019; Šabić et al., 2020; Stacey et al., 2020). Beyond gaze location, metrics such as fixation duration and saccade frequency offer further insight into cognitive load. Longer fixations and reduced gaze shifts are typically associated with increased processing difficulty, particularly in noisy environments (Cui and Herrmann, 2023; Šabić et al., 2020; Wendt et al., 2015). These behavioral changes often co-occur with physiological markers such as increased pupil dilation, reflecting listening effort and general arousal (Naylor et al., 2018). Together, these findings suggest that increased cognitive demands during speech comprehension in challenging listening conditions are reflected in alterations in eye movement behavior.

In face-to-face conversation, gaze serves a dual function: it supports the perceptual processing of speech and acts as a social signal to coordinate interaction (Risko et al., 2012, 2016). Gaze behavior plays a key role in managing turn-taking, as it communicates engagement and helps guide the flow of conversation. Talkers use gaze to monitor listener engagement, avert their eyes to hold the floor, or re-establish eye contact to signal turn-yielding (Degutyte and Astell, 2021; Kendrick et al., 2023). Listeners typically gaze at the talker to aid comprehension and signal attentiveness but may avert their gaze to avoid taking the turn or to process information more deeply (Degutyte and Astell, 2021; Oertel et al., 2015). Gaze is also involved in response planning: next talkers often begin formulating their response before the current turn ends, using prosodic and visual cues - such as gaze direction and gesture timing – as turn-end signals to time their own entry (Barthel et al., 2017). Talkers may direct their gaze toward a specific interlocutor to select them as the next talker or avoid eye contact to hold the floor (Auer, 2021; Degutyte and Astell, 2021). Gaze-based cues are especially important in multi-party conversations, where managing participation and talker transitions becomes more complex. Group size, task demands, and social context all influence gaze behavior, with larger groups requiring more effortful coordination (Degutyte and Astell, 2021; Maran et al., 2021), and dual-task conditions reducing gaze toward the talker (Hendrikse et al., 2019). Together, these findings underscore the predictive and regulatory role of gaze in conversational turn taking, where visual cues help anticipate turn boundaries and maintain interactional flow.

This dual function of gaze during conversations raises questions to what extent the findings about changes in eye movement from controlled laboratory settings with passive listening tasks can be generalized to face-to-face movement (Risko et al., 2012, 2016). Two recent studies have therefore investigated the impact of background noise on eye-movements during active participation in conversations (Hadley et al., 2019; Lu et al., 2021). Hadley et al. (2019) observed a shift in visual attention during communication, noting that as background noise increased, interlocutors spent more time looking at the talker’s mouth rather than their eyes. This shift suggests a re-prioritization of visual information, where listeners may rely less on the eyes - which typically conveys engagement and turn-taking cues - and instead focus more on the mouth to aid speech comprehension. Lu et al. (2021) examined eye-movements in triadic conversations and found that normal-hearing listeners increased their visual focus on the active talker as noise levels rose. In contrast, older or hearing-impaired individuals exhibited more distributed gaze patterns, potentially reflecting greater difficulty in predicting turn transitions or an increased need to scan the scene for useful visual cues. These studies represent early but important steps in understanding how acoustic environments shape visual attention in face-to-face communication and highlight the potential of eye-tracking as a tool for assessing communication difficulty in realistic settings.

However, the study by Hadley et al. (2019) focused on dyadic conversations, which differ conceptually from group conversations. In dyadic settings, attention is stable and clearly directed and turn-taking dynamics are less complex (Hadley et al., 2021; Maran et al., 2021; Moreland, 2010). While Lu et al. (2021) addressed this limitation by examining triadic conversations, their experimental setup involved the same two confederates interacting with each participant, potentially introducing biases in speech production. Moreover, their analysis averaged gaze behavior across entire conversations, thereby overlooking the dynamic role of gaze as an active tool for communication. It also remains unclear to what extent other eye-movement measures - such as fixation duration, saccade latency, and pupil dilation – which have previously been identified as indicators of listening difficulties in passive tasks (Cui and Herrmann, 2023; Hidalgo et al., 2022; Šabić et al., 2020; Van Engen and McLaughlin, 2018), can be reliably used to assess communication difficulties in more realistic conversational settings.

The present study aimed to investigate how gaze patterns adapt during triadic face-to-face conversations under challenging acoustic conditions. Specifically, we asked whether background noise alters the allocation of visual attention during conversation, and whether markers of cognitive load - such as fixation duration, saccade rate, and pupil dilation - reflect the increased effort required to maintain conversational flow in noise. By examining these questions in a realistic, interactive setting, we sought to bridge the gap between laboratory-based findings and the complexities of real-world communication. To address these questions in a realistic and interactive setting, we recorded eye movements and speech during structured triadic conversations in 10 groups, each comprising three normal-hearing participants unfamiliar with each other. Conversational difficulty was manipulated by introducing two levels of multi-talker babble. To link turn-taking behavior with gaze dynamics, we extracted cognitive load markers and compared them across acoustic conditions and between listening and speaking/talking segments.

## Materials and methods

### Participants

Thirty young, native Danish-speaking participants (aged 20–30 years) were organized into 10 triads, consisting of six mixed-gender and four single-gender groups. Within each triad, participants were not acquainted with one another. All participants had normal or corrected-to-normal vision, self-reported normal hearing, and no medical conditions that could potentially affect their communication abilities. Prior to the experiment, participants provided written informed consent and were financially compensated for their involvement afterwards. The experimental procedure was approved by the Science Ethics Committee for the Capital Region of Denmark (reference H-16036391).

### Task and procedure

**Figure 1:**
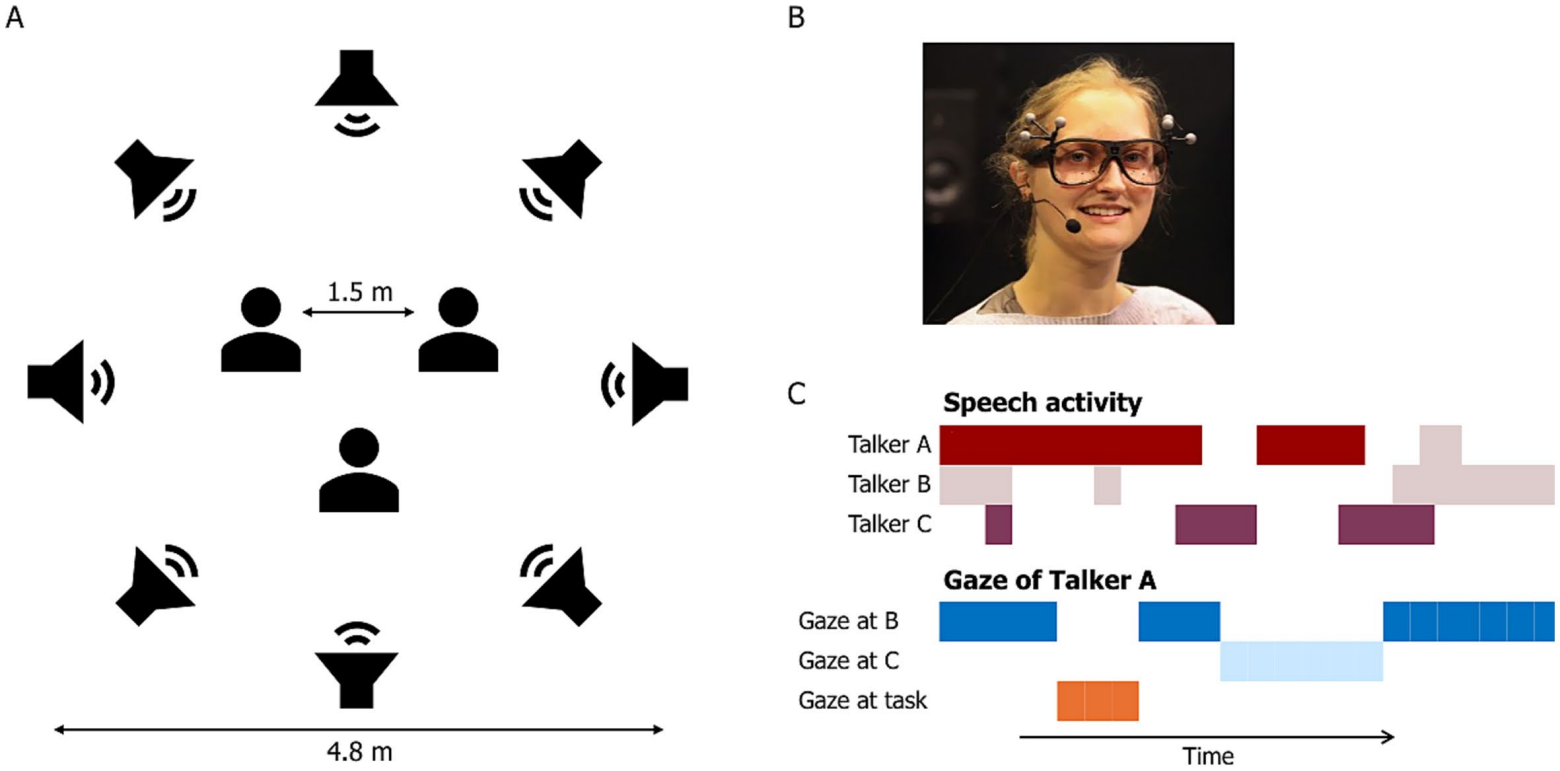
Experimental set-up. **(A)** A ring of eight loudspeakers presented 8-talker babble noise during each trial, with three participants seated in the center of the ring. **(B)** Each participant was equipped with eye-tracking glasses, a close-mouth microphone, and two in-ear microphones. **(C)** Speech activity for each participant was detected and synchronized with their gaze location and behavior.

Participants were seated in a triangular arrangement, each 1.5 meters apart, at the center of a ring of 16 equidistant loudspeakers with a 2.4-m radius (Figure 1A). A black curtain surrounded the loudspeaker ring to reduce visual distractions in the conversation area. Eight of the loudspeakers played different Danish monologues spoken by different talkers, taken from the corpus described in Lund et al. (2019), creating an 8-talker babble noise (Figure 1A). The monologues, each lasting approximately 90 s, were looped continuously throughout the conversation. The loudspeakers were powered by a sonible d:24 amplifier (sonible GmbH, Austria). Background noise was presented at sound levels of 48 dB for the ‘quiet’ condition and 78 dB for the ‘noise’ condition (Hadley et al., 2019, 2021).

Each participant was equipped with three microphones to record their speech (Figure 1B): a close-mouth microphone (DPA 4288, DPA Microphones, Denmark) and two in-ear microphones (DPA 4560, DPA Microphones, Denmark), resulting in a 9-channel recording for each conversation. Eye movements were recorded using Tobii Pro Glasses 3 (Danderyd, Sweden) with a sampling frequency of 50 Hz. The glasses also recorded a scene video at 25 Hz (field of view: 95 deg. × 63 deg., resolution: 1920 × 1,080). All recordings from the Tobii glasses were stored locally on an SD card within the recording unit and streamed to a computer (running Windows 10) through the Tobii Pro Glasses 3 controller app, allowing real-time monitoring of the recording process.

The experiment was controlled using MATLAB, which managed the playback of the 8-channel background noise and the recording of the 9-channel speech signals at a 48 kHz sample rate. The MATLAB script also triggered eye-tracking via a USB connection between the computer and the recording unit of the Tobii glasses. To synchronize the audio data recorded by the Tobii glasses’ built-in microphone with the eye-tracking data, the maximum cross-correlation and corresponding delay between the two audio streams were calculated (Lu et al., 2021).

Before the experiment began, participants were introduced to the study procedure and given time to get acquainted with each other. This period varied slightly across groups due to differences in arrival times but typically lasted around 10 min. They were then moved to the laboratory and seated in a triangular arrangement (Figure 1A). Each participant was fitted with microphones and eye-tracking glasses, which were individually calibrated using Tobii’s one-point calibration procedure before the start of each recording session.

Each trial consisted of three phases, with participants performing an item-comparison task both before and after a conversation centered around the task (Örnolfsson et al., 2024). This task was chosen to ensure participant engagement in the conversation while maintaining control over the topic. There were 8 sets, each containing 8 items, across four topics: tech companies, movies, countries, and landmarks in Copenhagen. During the item-comparison task, participants individually evaluated all possible pairwise combinations of items, judging their order (e.g., by year, population size, or distance) on a scale from – 100 to 100, which also reflected their confidence in their judgment. Items were presented, and responses were recorded using a Unity app running on touch-screen tablets.

After the initial comparison task, participants entered the communication phase, during which they discussed the item order. A paper sheet displaying all items from the set was placed in front of each participant on a holder. The experimenter initiated this phase by clapping (to assist with audio alignment), followed by the playback of background noise. Participants had up to 10 min to discuss the items and signaled to the experimenter when their discussion was complete. Following the communication phase, participants answered five questions via the Unity app, rating aspects such as listening effort, engagement, perceived conversation fluency, speaking difficulty, and speech understanding on a scale from 0 to 10 (see Table 1). They then performed the item-comparison task again. Communication was only allowed during the discussion phase, not during individual judgements. The presentation of item pairs and the order of questions were randomized. Each topic was presented in two acoustic conditions (one in noise and one in quiet), with the order of topics and noise conditions randomized.

**Table 1:** Question items, dimensions, and verbal markers of scale endpoints for ratings performed after each conversation.

Dimension	Question	Scale (0–10)	
Listening effort	Did you have to put in a lot of effort to hear what was being said in the conversation?	No effort	A lot of effort
Speech understanding	How well did you understand what the others were saying?	Not at all	Extremely well
Speaking difficulty	How easy was it for you to express what you wanted to say during the conversation?	Very difficult	Very easy
Engagement	How engaged were you to actively participate in the conversation?	Unengaged	Very engaged
Conversational flow	How was the flow of the conversation?	Very poor flow	Good flow

The table lists the question items used for post-conversation ratings, the corresponding dimensions they assess, and the verbal markers indicating the scale’s endpoints for ratings of 0 and 10, respectively.

Before the actual experiment, triads completed two full practice rounds using the tech company item sets to familiarize themselves with the task. Participants were given a short break after approximately 1 h, and the entire experiment lasted around 2.5 h.

### Speech recordings and segmentation

Each participant was equipped with a cheek-mounted directional microphone (DPA 4288, DPA Microphones, Denmark) and a pair of in-ear microphones (DPA 4560, DPA Microphones, Denmark). The nine microphones were connected to two pre-amplifiers (RedNet MP8R, Focusrite), and speech was recorded at a sampling frequency of 48 kHz using MATLAB (R2021b).

The 9-channel audio recorded during each conversation task was segmented using offline voice activity detection (VAD) for each talker, as illustrated in Figure 1C. VAD consisted of two stages, an energy-based identification of speech activity for each talker and a cross-correlation stage to eliminate crosstalk. The audio signal was preprocessed using a high-pass filter with a cut-off frequency at 100 Hz to remove breathing noise. The filtered audio signal was then divided into 20 ms frames using rectangular windows with 50% overlap. For each frame, the root-mean-square (RMS) was calculated for each individual channel, similar to the method described by Beechey et al. (2018). The frame-based RMS from the close-mouth microphones exhibited a bimodal distribution, with one peak corresponding to background noise and the other to the talker’s speech. These distributions were used to define an RMS threshold for identifying speech regions for each conversation.

If speech activity is detected because of crosstalk, this will be both visible in an RMS level difference and the cross correlation. If more than one person is talking at the same time, the RMS level for all close-mouth microphones is above the threshold and the RMS level difference is low. Therefore frames, where a participant’s RMS level was more than 6 dB lower than that of another talker, were classified as crosstalk (Beechey et al., 2018). Another marker for crosstalk is the cross correlation between the microphones. The lag corresponding to the maximum cross-correlation provides insight into the signal’s origin relative to the microphones. If a signal originates closer to a participant’s close-mouth microphone than the ones of the other participants, the lag between their own microphone and those of the other talkers will be negative. For the application of the cross-correlation threshold, the maximum cross-correlation and corresponding lag between channels were computed, constrained to a lag range of −300 and + 300 samples. Frames, where the RMS difference was less than 6 dB were retained only if the lag of the cross-correlation between the participant’s microphones was negative, indicating that the signal originated closer to their microphones. To further filter out noise, we excluded segments where the maximum cross-correlation delay between a participant’s close-mouth and in-ear microphones was below 0.8 ms, corresponding to a distance of approximately 30 cm.

Following this, speech intervals with gaps shorter than 180 ms were merged, and intervals shorter than 90 ms were removed, as described by Heldner and Edlund (2010). Utterances detected for each talker were then combined into turns by merging uninterrupted pauses within the same talker. Turns shorter than 1 s were excluded, as they likely contained noise or vocal backchannels, such as ‘yes’, ‘ok’, ‘mmm’, or ‘nå’ (Hadley and Culling, 2022). This segmentation process resulted in precise timestamps for the onset and offset of each turn for each talker, enabling detailed analysis of their conversational dynamics.

### Eye-movement analysis

The Tobii 3 glasses recorded pupil position and diameter, gaze origin and direction for each eye, as well as gaze position in both 3D and 2D formats. Raw data were processed and analyzed using MATLAB (R2023a). Trials with more than 30% missing data were excluded from the analysis. Blinks were detected based on binocular missing data and pupillometry noise (Hershman et al., 2018). A time constraint was applied, defining a blink as having a duration between 50 ms and 1,000 ms (Brych et al., 2021). Pupil and gaze data during blinks were removed. Eye direction data for each eye, along with the 2D data, were converted into angular direction data, which were then used to calculate angular velocity. Data with angular velocities exceeding 1,000 deg./s or with zero position were discarded. Gaze location data were smoothed using a 5th order Savitzky–Golay filter with a frame length of 9 (Mack et al., 2017), and missing data gaps shorter than 200 ms were linearly interpolated. Pre-processed gaze data were classified into fixations and saccades (rapid eye movements between fixation points) using an I-DT algorithm with a dispersion threshold of 1.5 degrees (Salvucci and Goldberg, 2000) and a minimum duration of 100 ms. Pupil data were smoothed using a moving median filter of 200 ms and were linearly interpolated. To account for potential differences in luminance and gaze location effects, baseline pupil dilation was calculated separately for each face, using the average pupil dilation during the longest fixation on each face prior to noise onset. Only baseline-subtracted pupil data recorded while participants were gazing at their interlocutors’ faces were included in the analysis.

Areas of interest (AOIs) for the interlocutors, their faces, and the task materials were identified in each video frame using Python. People detection was performed using YOLOv5n (Jocher et al., 2022), face detection with MediaPipe (Lugaresi et al., 2019), and task material detection with OpenCV’s ORB detector (Rublee et al., 2011) paired with a FLANN matcher for feature mapping. The AOI data obtained were spatially post-processed to remove outliers by applying a moving median filter with a window size of 3 to the coordinate and size vectors. AOI dimensions were refined, with face AOIs ranging between 50 and 300 pixels, people AOIs between 50 and 900 pixels, and task material AOIs between 100 and 1,400 pixels. Temporal post-processing was applied by linearly interpolating gaps of fewer than 50 frames to reduce false negative detections. Short occurrences lasting fewer than 4 frames were treated as false positives (De Beugher et al., 2014). AOIs were enlarged by 40 pixels (approximately 2 degrees visual angle) to account for the Tobii glasses’ accuracy (1.60 + −0.98 deg., Onkhar et al., 2023). Finally, 2D gaze position data were linearly interpolated to annotate gaze points within an AOI.

**Figure 2:**
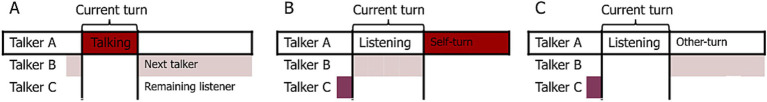
Roles at the offset of talking and listening from Talker A’s perspective. **(A)** Talker A is talking during the current turn, with Talker B designated as the next talker and Talker C remaining as the listener. **(B)** When Talker A is listening during the current turn, they can take the next turn themselves, referred to as a “self-turn.” **(C)** Alternatively, Talker A can remain listening while another participant, such as Talker B, takes the next turn, referred to as an “other-turn.”

From the processed eye-tracking data, we extracted a set of features previously associated with cognitive load and conversational turn-taking (Degutyte and Astell, 2021; Mahanama et al., 2022). During periods of talking and listening, we calculated the average fixation duration and saccade rate. Additionally, we measured the average and maximum changes in pupil dilation from baseline for these periods. To describe the overall distribution of visual attention throughout each conversation, we calculated the dwell time for each AOI. Gaze location and timing, relative to the onset and offset of listening and talking, were analyzed to investigate their roles in managing turn-taking dynamics. At the offset of listening, we distinguished between two scenarios: when the current listener becomes the next talker (‘self-turn’, Figure 2B) and when another listener takes the next turn (‘other-turn’, Figure 2C). Similarly, at the offset of talking, we compared the gaze behavior of the current talker directed towards the listener who becomes the next talker (‘next talker’) with that directed towards the listener who continues to listen (‘remaining listener’, Figure 2A).

### Statistical analysis

We employed mixed-effect models to analyze the impact of noise and conversational state on the various outcome measures (y). Unless otherwise specified, the baseline model prior to reduction was structured as: y ~ noise condition * conversation state + (1|group/participant). Models were constructed in *R* using the *lme4* package (Bates et al., 2015). Stepwise model reduction was subsequently performed on the baseline model using the *lmerTest* package (Kuznetsova et al., 2017). The effects of the remaining factors in the reduced model were analyzed using analysis of variance. *Post hoc* within-factor analyses were conducted using the *emmeans* package (Lenth, 2023). Effect sizes are reported as partial η^2,^ calculated using the *effectsize* package (Ben-Shachar et al., 2020).

## Results

### Gaze location

**Figure 3:**
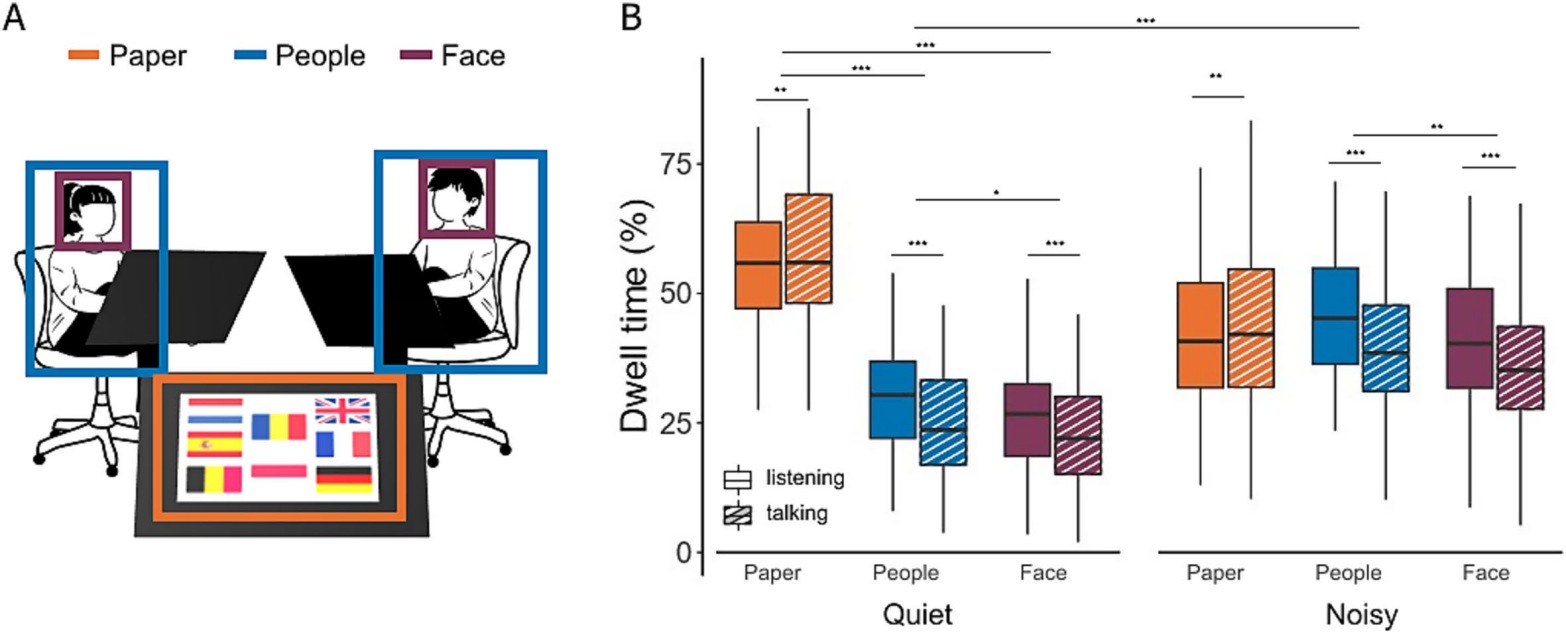
Gaze location during conversations in quiet and noisy conditions. **(A)** Areas of interest (AOIs), including task material (orange), interlocutors (blue), and the faces of interlocutors (purple). **(B)** Comparison of gaze distributions in quiet and noisy environments. Dwell time represents the percentage of time participants directed their gaze towards the different AOIs during both listening (filled bars) and talking (hatched bars) phases. Box plots show the median, first and third quartiles, with whiskers representing the minimum and maximum values. **p < 0.01, ***p < 0.001.

The distribution of the gaze location during a conversation can serve as an indicator of visual attention focus. We defined three AOIs in the scene videos: other participants, their faces, and the task material. Figure 3 illustrates the proportion of time participants directed their gaze at each AOI during periods of both listening and talking.

In quiet conditions, participants spent significantly more time looking at the task material compared to their interlocutors [28.9%, *t*(852) = 19.04, *p* < 0.0001] or their faces [31.9%, *t*(852) = 20.98, *p* < 0.0001]. A small but significant main effect of conversation state and a large interaction effect between conversation state and AOIs were observed (Table 2). Post-hoc tests revealed that the effect of conversation state was primarily driven by differences in the face and people AOIs. Specifically, participants exhibited a 5.7% increase in dwell time on faces during listening compared to talking [*t*(854) = 3.62, *p* = 0.0003] and a 6.5% increase in dwell time on the people AOI [*t*(854) = 4.10, *p* < 0.0001]. No significant difference in dwell time on the task material was observed between listening and talking [*t*(854) = −2.42, *p* = 0.02]. In noisy conditions, gaze behavior shifted notably. Dwell time on the task material decreased by 16.4% [*t*(860) = 10.33, *p* < 0.0001], while gaze time on interlocutors increased by 15.6% [*t*(860) = −9.86, <0.0001]. As a result, there was no longer a significant difference in gaze time between the task material and interlocutors in noisy environments [*t*(852) = −1.87, *p* = 0.06].

**Table 2:** Statistical results for all analyzed outcome measures.

Feature	Fixed effect	F-statistics	*p*-value	Effect size
Dwell time	Noise condition	*F*(1,879.25) = 23.78	<0.001	0.03
	Conversation state	*F*(1,863.16) = 9.39	0.002	0.01
	AOI	*F*(2,857.42) = 121.75	<0.001	0.22
	Noise condition: AOI	*F*(2,857.42) = 130.43	<0.001	0.23
	Conversation state: AOI	*F*(2,857.42) = 13.62	<0.001	0.03
Mean pupil dilation	Noise condition	*F*(1,291.23) = 11.00	0.001	0.04
Maximum pupil dilation	Noise condition	*F*(1,292.24) = 16.61	<0.001	0.05
Mean fixation duration	Noise condition	*F*(1,272.48) = 18.72	<0.001	0.06
	Conversation state	*F*(1,268.22) = 109.22	<0.001	0.29
	Noise condition: Conversation state	*F*(1,268.22) = 7.12	0.008	0.03
Saccade rate	Noise condition	*F*(1,272.36) = 13.38	<0.001	0.05
	Conversation state	*F*(1,268.74) = 9.19	0.003	0.03
	Noise condition: Conversation state	*F*(1,268.74) = 8.90	0.003	0.03
Gaze at onset				
Listening: Median gaze latency talker	Noise condition	*F*(1,126.98) = 45.74	<0.001	0.26
Listening: IQR gaze latency talker	Noise condition	*F*(1,126.99) = 1.44	0.23	<0.01
Listening: First fixation on talker	Noise condition	*F*(1,124.33) = 74.33	<0.001	0.37
Talking: Median gaze latency people	Noise condition	*F*(1,129.26) = 19.46	<0.001	0.13
Talking: IQR gaze latency people	Noise condition	*F*(1,130.14) = 16.26	<0.001	0.11
Talking: First fixation on people	Noise condition	*F*(1,125.08) = 45.27	<0.001	0.27
Gaze at offset				
Listening: Median gaze latency talker	Noise condition	*F*(1,300.36) = 5.88	0.02	0.02
	Next turn	*F*(1, 297.22) = 14.12	<0.001	0.05
	Noise condition: Next turn	*F*(1,297.22) = 5.88	0.02	0.02
Listening: Last fixation on talker	Noise condition	*F*(1,285.70) = 53.77	<0.001	0.16
	Next turn	*F*(1, 279.51) = 9.43	0.002	0.03
Listening: Dwell time talker – other listener	Noise condition	*F*(1,287.5) = 47.13	<0.001	0.14
	Next turn	*F*(1, 279.4) = 22.83	<0.001	0.08
Talking: Median gaze latency people	Next turn	*F*(1, 307) = 14.45	<0.001	0.04
Talking: Last fixation on people	Noise condition	*F*(1,284.39) = 53.28	<0.001	0.16
	Next turn	*F*(1,278.31) = 174.86	<0.001	0.39
Talking: Dwell time people	Noise condition	*F*(1,283.31) = 61.25	<0.001	0.18
	Next turn	*F*(1,277.80) = 150.83	<0.001	0.35
	Noise condition: Next turn	*F*(1,277.80) = 6.29	0.01	0.02

### Gaze at onset

**Figure 4:**
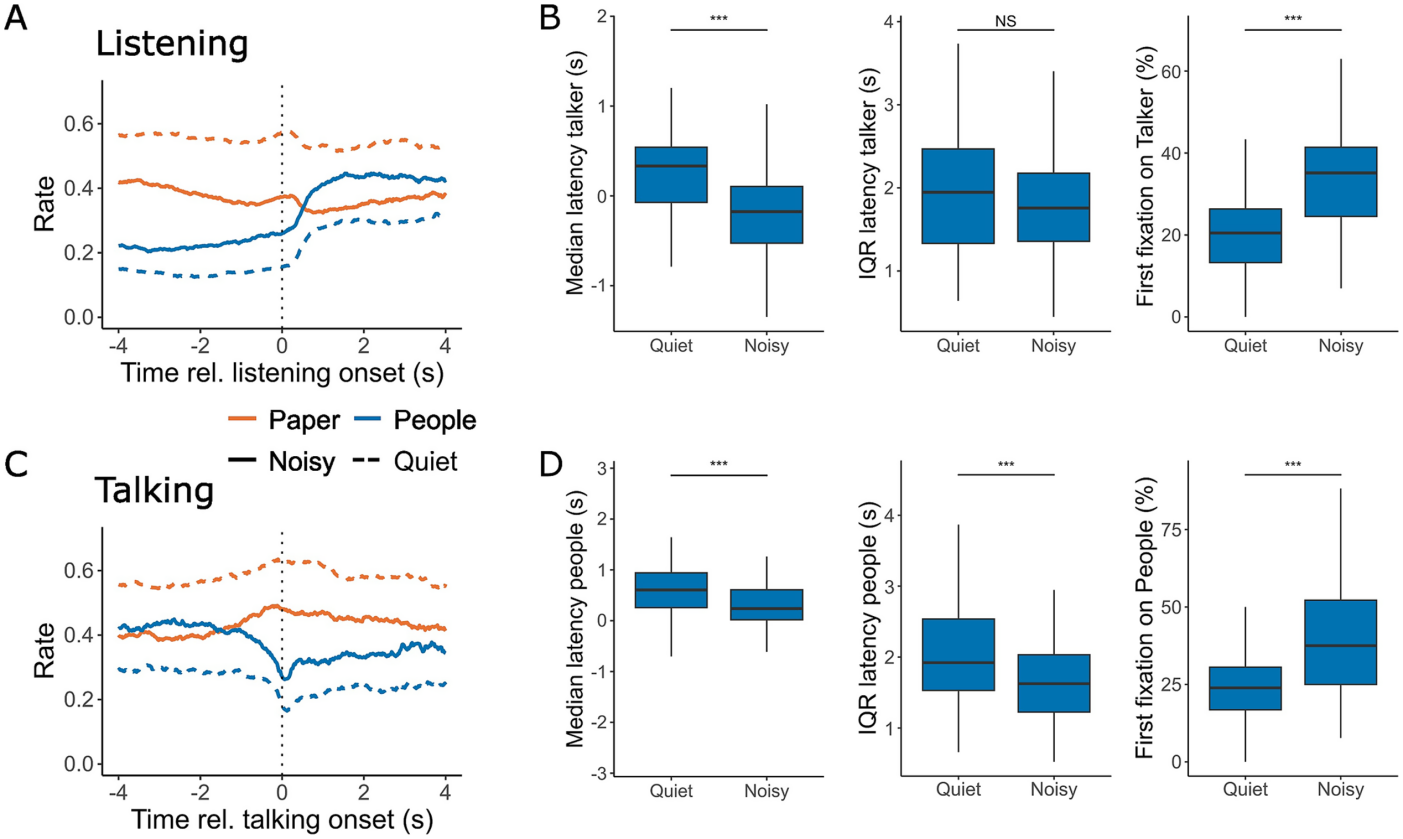
Gaze location at the onset of listening **(A,B)** and talking **(C,D)**. **(A,C)** The rate at which gaze was directed toward the task material (orange) and interlocutors (blue) within a 4-s window before and after the onset of listening **(A)** and talking **(C)** during conversations in quiet (dashed line) and noisy (solid line) conditions. **(B,D)** The corresponding median and IQR of the latency for looking at the current talker **(B)** and listeners **(D)**. Panels also display the likelihood of the first fixation after turn onset being directed toward the talker (**B**, right) or one of the listeners (**D**, right). Box plots represent the median, first and third quartiles, with whiskers indicating the minimum and maximum values. **p < 0.01, ***p < 0.001.

We analyzed gaze behavior at the onset of a turn during both listening and talking. Participants exhibited a slight increase in looking at the task material both just before and after the onset of a turn. Additionally, they looked towards the current talker more frequently following the onset of listening (Figure 4A). Background noise had a significant effect on the median latency with which participants directed their gaze to the current talker [*F*(1,126.98) = 45.74, *p* < 0.001, Table 2], with gaze shifts occurring 424 ms earlier in noisy conditions compared to quiet conversations (Figure 4B). However, noise did not significantly affect gaze latency variability, as measured by the interquartile range (IQR) of the latency [*F*(1,126.99) = 1.44, *p* = 0.23]. Additionally, the first fixation was directed to the current talker 14% more often during noisy conversations [*F*(1,124.33) = 74.33, *p* < 0.001], consistent with the overall increase in gaze towards interlocutors observed throughout conversations in noisy conditions.

After taking a turn, talkers more frequently shifted their gaze to the task material in both quiet and noisy environments (Figure 4C). Noise had a moderate effect on both the median latency of these gaze shifts [*F*(1,129.26) = 19.46, *p* < 0.001] and the IQR of the latency [*F*(1,130.14) = 16.26, *p* < 0.001], as shown in Figure 4D. Furthermore, the probability that the first fixation was directed toward interlocutors was 16% higher in the noisy conditions compared to quiet [*F*(1,126.83) = 47.717, *p* < 0.001]. Overall, gaze patterns appeared to be more synchronized with the onset of turns in noisy conversations, suggesting that noise increases the attentional coordination of visual behavior during turn taking.

### Gaze at offset

**Figure 5:**
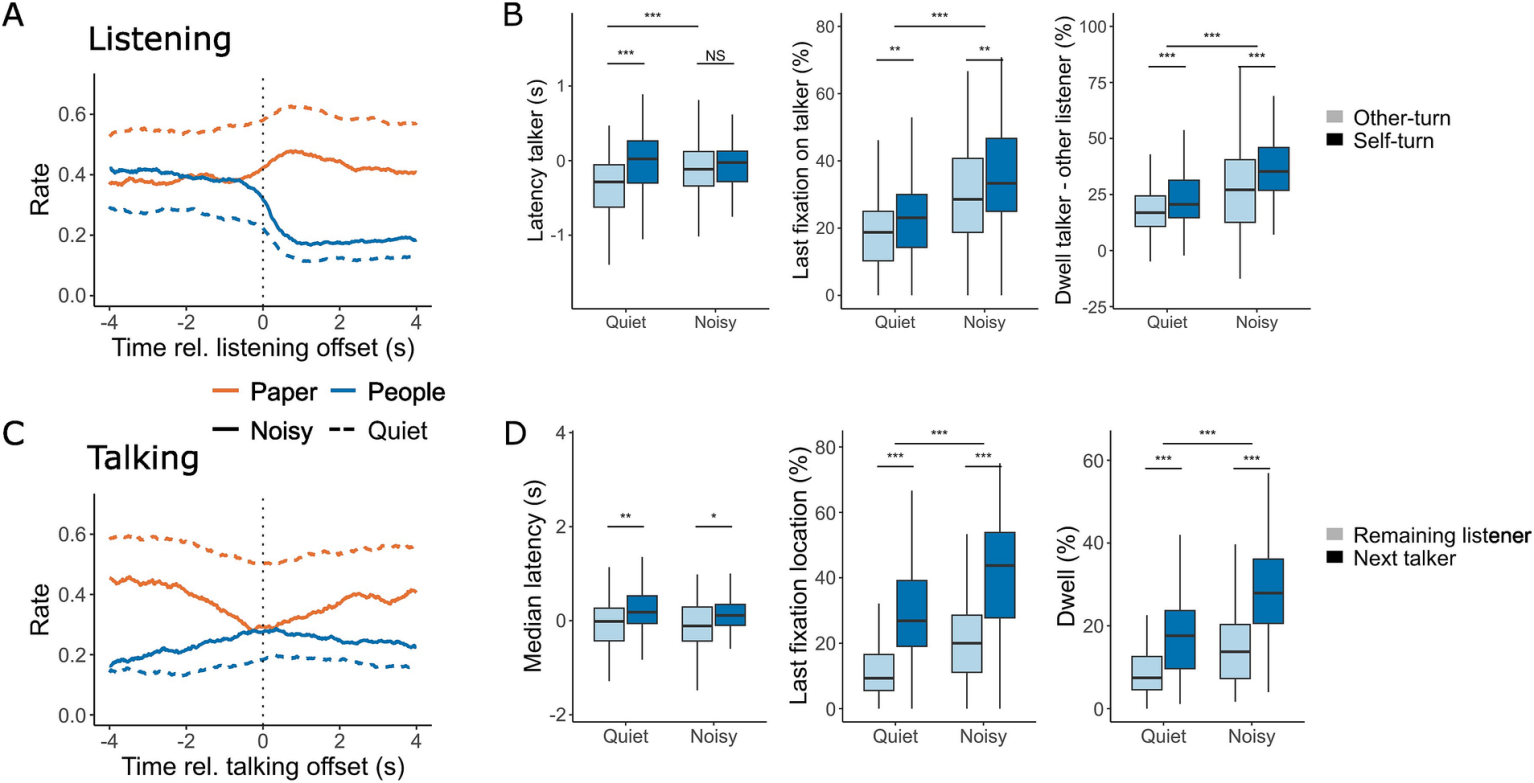
Gaze location at the offset of listening **(A,B)** and talking **(C,D)**. **(A,C)** The rate at which gaze was directed towards the task material (orange) and interlocutors (blue) within a 4-s window before and after the offset of listening **(A)** and talking **(C)** during conversations in quiet (dashed line) and noisy (solid line) conditions. **(B,D)** The corresponding median latency for looking at the current talker (**B**, left) and listeners (**D**, left), as well as the last fixation directed toward the talker (**B**, middle) and listeners (**D**, middle) during quiet and noisy conversations. In **(B)** (right), the difference in dwell time between gazing at the current talker and the other listener is shown, with positive values indicating more time spent looking at the current talker. In **(D)** (right), dwell time directed at listeners during a talking turn is shown for both quiet and noisy conditions. In **(B)**, light blue shading represents instances where the current listener remains listening while the other listener takes the turn, and dark blue shading represents a self-turn, where the current listener becomes the next talker. In **(D)**, light blue shading indicates gaze directed towards the listener who remains listening, while dark blue shading indicates gaze directed towards the listener who becomes the next talker. Box plots display the median, first, and third quartiles, with whiskers indicating the minimum and maximum values. *p < 0.05, **p < 0.01, ***p < 0.001.

Figure 5 shows the gaze patterns at the offset of both listening (Figures 5A,B) and talking (Figures 5C,D). At the end of listening, participants shifted their eye gaze away from their interlocutors and towards the task material. When participants were taking the next turn, they shifted their gaze 313 ms later compared to when the other listener was taking the turn [*F*(1, 297.22) = 14.12, *p* < 0.001], resulting in better alignment between gaze and the turn offset. A small effect of noise was observed [*F*(1,300.36) = 5.88, *p* = 0.02], with gaze shifts occurring 246 ms later in noisy conversations compared to quiet ones. Additionally, a significant interaction between background noise and the identity of the next talker was found [*F*(1,297.22) = 5.88, *p* = 0.02]. Post-hoc tests revealed that this interaction was primarily driven by changes in gaze latency when the other listener was taking the turn [*t*(299) = −3.43, *p* = 0.0007]. No significant difference was observed between noise conditions when the listener themselves was taking the turn [*t*(299) = −0.002, *p* = 1.00].

In noisy conversations, the last fixation before the end of listening was more likely to be directed toward the current talker [*F*(1,283.47) = 53.12, *p* < 0.001, Table 2], and this likelihood slightly increased when participants were about to take the next turn [*F*(1, 277.54) = 9.27, *p* = 0.003]. During listening, participants gazed more at the talker than at the other listener, with this effect being more pronounced in noisy conditions (*F*(1,285.26) = 46.85, p < 0.001), particularly when the listener was preparing to take the next turn [*F*(1, 277.54) = 23.01, *p* < 0.001]. These findings suggest that gaze patterns during listening provide cues about whether a listener is preparing to take the next turn.

At the end of talking, participants increased their gaze towards their interlocutors and reduced their gaze towards the task material (Figure 5C). A small but significant increase in the latency of gaze shifts directed toward the next talker compared to the other listener was observed [*F*(1, 307) = 14.45, *p* < 0.001]. The final fixation before the end of talking was more frequently directed at the interlocutor who would take the next turn rather than the one who would continue listening [*F*(1,276.28) = 173.50, *p* < 0.001]. Noise had a large effect on the likelihood of the final fixation being directed at an interlocutor [*F*(1,282.11) = 52.78, *p* < 0.001], reflecting the overall tendency to focus more on people during noisy conversations. Talkers spent more time looking at the next talker compared to the other listener [*F*(1,275.85) = 149.84, *p* < 0.001], with an overall increase in gaze duration towards interlocutors in noisy conditions [*F*(1,281.10) = 60.26, *p* < 0.001]. A significant interaction effect [*F*(1,275.85) = 6.09, *p* = 0.01] revealed that the difference in gaze duration was larger in noise, at 14.4% [*t*(276) = 10.1, *p* < 0.001], compared to 9.5% in quiet [*t*(276) = 7.1, *p* < 0.001]. These patterns suggest that the talker’s gaze behavior provides important cues about who will take the next turn, with gaze dynamics being more pronounced and coordinated in noisy conversations.

### Eye movement and pupillometry

**Figure 6:**
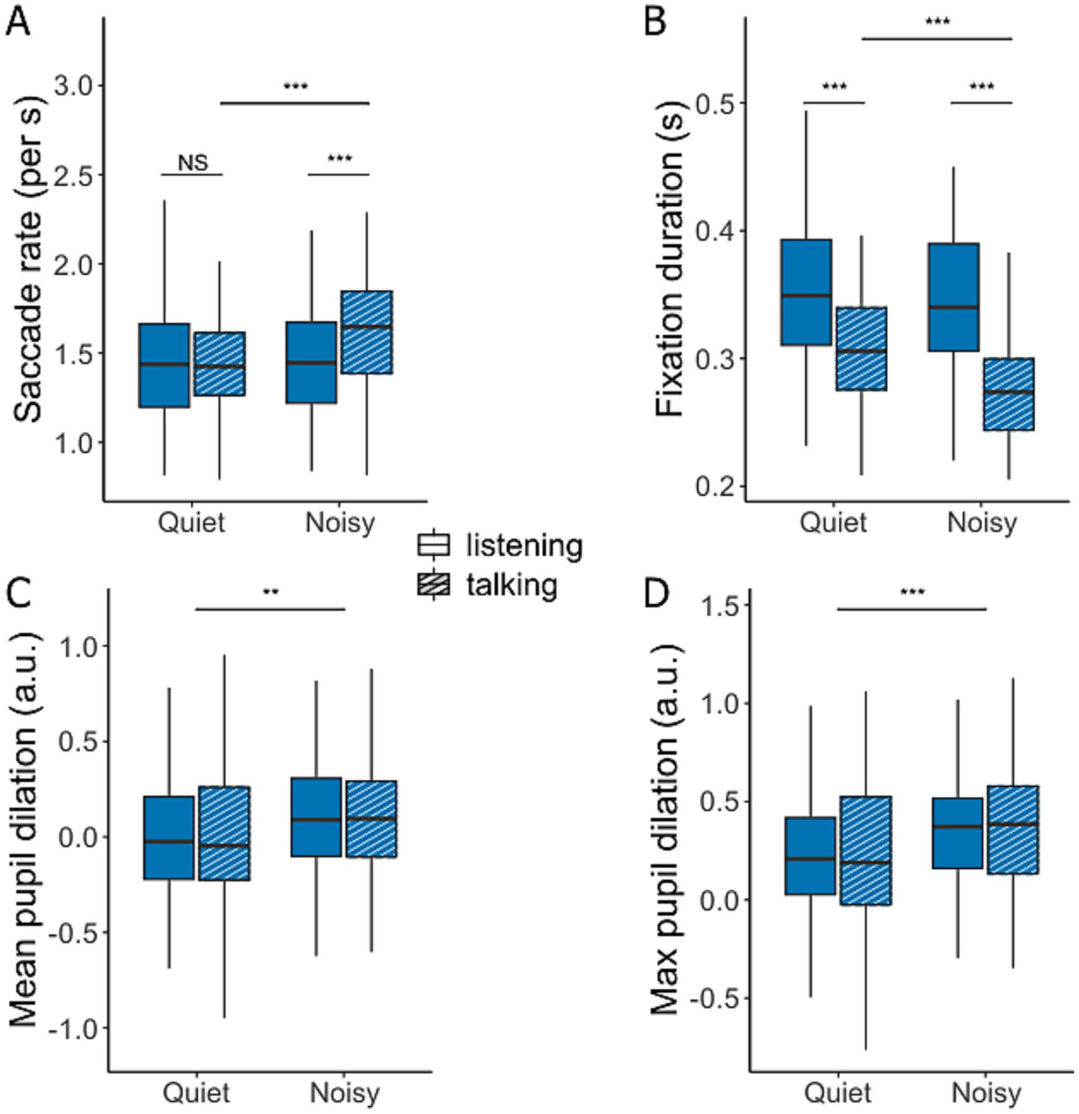
Saccade rate **(A)**, mean fixation duration **(B)**, and mean **(C)** and maximum pupil dilation **(D)** during conversations in quiet and noisy conditions. Data are presented for talking (hatched bars) and listening (filled bars). Box plots display the median, first, and third quartiles, with whiskers indicating the minimum and maximum values. *p < 0.05, **p < 0.01, ***p < 0.001.

Figure 6 illustrates the differences in eye movements and pupil dilation between quiet and noisy background conditions during periods of listening and talking. The saccade rate (Figure 6A) was significantly higher when participants conversed in noise compared to quiet conditions [*F*(1,272.36) = 13.38, *p* < 0.001] and while talking compared to listening [*F*(1,268.74) = 9.19, *p* = 0.003]. A significant interaction effect [*F*(1,268.74) = 8.90, *p* = 0.003] revealed a pronounced difference between listening and talking during noisy conditions [*t*(269) = −4.16, *p* < 0.0001], whereas no significant was observed in quiet conditions [*t*(269) = −0.03, *p* = 0.97].

A complementary pattern was observed in the average fixation duration (Figure 6B), which significantly decreased during talking compared to listening [*F*(1,268.22) = 109.22, *p* < 0.001] and in noisy compared to quiet conditions [*F*(1,272.48) = 18.72, *p* < 0.001]. A significant interaction between background noise and conversation state [*F*(1,268.22) = 7.12, *p* = 0.008] indicated that fixation duration significantly decreased during talking in noisy conditions [*t*(270) = 4.95, *p* < 0.0001]. However, the reduction in fixation duration during listening in noisy versus quiet conversations was not significant [*t*(270) = 1.20, *p* = 0.23].

Pupil dilation, a common marker of cognitive load and listening effort, showed no significant effect based on the conversation state (Figures 6C,D). Nevertheless, participants exhibited an increased average pupil dilation [*F*(1,291.23) = 11.00, *p* = 0.001] and maximum pupil dilation [*F*(1,292.24) = 16.61, *p* < 0.001] in noisy conditions compared to quiet. Although these effects were statistically significant, the effect sizes were small.

### Subjective ratings

**Figure 7:**
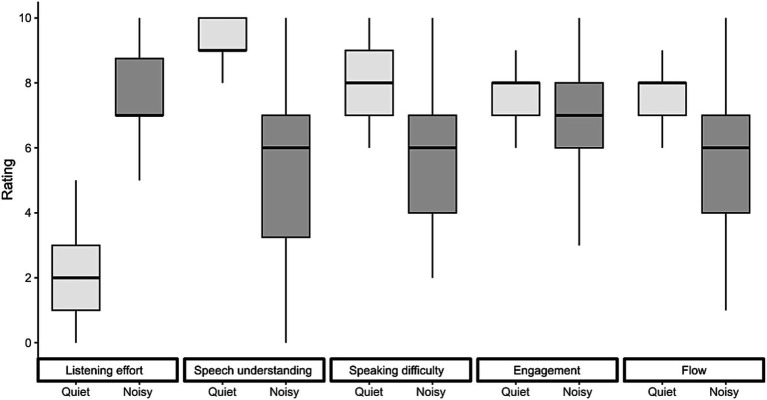
Subjective ratings of listening effort, speech understanding, speaking difficulty, engagement, and conversational flow. Ratings are shown for each conversation. Box plots display the median, first, and third quartiles, with whiskers indicating the minimum and maximum values.

The subjective ratings collected after each conversation are presented in Figure 7. All ratings were significantly affected by the presence of background noise. Conversing in noise resulted in a 5.4-point increase in perceived listening effort [*F*(1,149) = 732.07, *p* < 0.001], a 3.8-point decrease in speech understanding [*F*(1,149) = 284.32, *p* < 0.001], a 2.8-point decrease in speaking difficulty [*F*(1,149) = 141.68, *p* < 0.001], a 1.0-point decrease in engagement [*F*(1,149) = 14.35, *p* < 0.001], and a 1.9-point reduction in conversational flow [*F*(1,149) = 83.38, *p* < 0.001].

## Discussion

In this study, we investigated eye-movement behavior in relation to turn-taking during face-to-face communication and examined how background noise influences this behavior. Our goal was to determine whether noise-induced challenges alter the allocation of visual attention and whether the cognitive load required to maintain conversational flow is reflected in eye movement markers typically associated with listening difficulties in controlled laboratory settings. We found that under challenging acoustic conditions, participants directed their gaze more frequently toward their interlocutors rather than the task material (Figure 3). Gaze behavior also became more closely aligned with turn-taking dynamics in noisy environments, making it more predictive of the next talker (Figures 4, 5). While listening, gaze was focused on the talker’s face, while talking was associated with increased gaze movement. Markers previously linked to cognitive load showed either effects opposite to expectations or only small effect sizes in response to acoustic challenges (Figure 6). Subjective ratings (Figure 7) confirmed that a higher level of background noise made participating in conversations more difficult, reducing conversational flow and engagement.

### Effect of noise

In quiet settings, participants primarily directed their gaze toward the task material, using it both to prepare their next turn and to support short-term memory retention of discussed items. This behavior aligns with previous findings that individuals often offload cognitive demands by relying on readily available visual information in their environment (Ballard et al., 1995; Droll and Hayhoe, 2007; O’Regan, 1992). By looking at the task material, participants could reduce the need to retain information internally, thereby conserving cognitive resources. However, as background noise increased and communication became more challenging, participants shifted their gaze toward their interlocutors, sacrificing the memory support provided by the task material. This shift suggests that, under acoustically challenging conditions, participants allocate more cognitive resources to speech comprehension and rely more on visual cues to compensate for degraded auditory information. Previous studies have reported similar increases in visual attention to cues that support speech comprehension. For example, during passive listening tasks, individuals increased their gaze toward the talker’s mouth in noisy environments (Buchan et al., 2008; Stacey et al., 2020), and a similar trend was observed in active dyadic conversations (Hadley et al., 2019). Compared to Lu et al. (2021), who examined eye movements in triadic conversations under noise, the present study found a more pronounced shift in gaze toward interlocutors. This may be due to the inclusion of task material in our study, which added a competing area of interest. These findings underscore the sensitivity of gaze behavior to both visual scene complexity and conversational context. The increased reliance on visual cues in noise may also help explain why physical strategies to enhance audibility - such as turning the head or moving closer - often show limited effects. Such actions may conflict with social norms, whereas visual engagement serves both informational and social functions. This supports the notion of a multimodal Lombard effect, wherein visual and auditory strategies are integrated to maintain effective communication under challenging conditions (Dohen and Roustan, 2017; Trujillo et al., 2021).

Additionally, participants’ gazes aligned more closely with turn-taking behavior when communicating in noise compared to quiet conditions. Not only did listeners increase their total gaze duration toward the talker, but they also shifted their gaze to the talker earlier at the start of a turn change. Furthermore, gaze location at the end of a turn was more predictive of the next talker in noisy conditions than in quiet, for both listening and talking. This contrasts with findings from Hidalgo et al. (2022), who observed delayed gaze shifts when participants watched videos of conversations with vocoded speech, where greater speech degradation resulted in later gaze shifts. In recorded conversations, participants may not need to quickly reorient to the new talker, allowing them to prolong their gaze at the current talker to maximize visual information uptake. This is particularly beneficial with vocoded speech, which provides fewer acoustic cues to signal turn transitions. However, in active conversations, gaze also serves a social function (Risko et al., 2012, 2016), and lingering too long on the current talker may signal a desire to take the next turn (Degutyte and Astell, 2021). Our findings also differ from those of Hadley and Culling (2022) who studied head movements during triadic conversations and found greater variability in timing during noisy communication but no change in the average delay. This discrepancy may be because head movements, unlike gaze shifts, require more motor preparation and are energetically costly. Consequently, participants may be more cautious and deliberate in initiating head movements, waiting for clearer turn-transition cues.

In controlled laboratory studies where participants watch recorded conversations, gaze shifts tend to decrease, and fixations on the talker increase, suggesting an increased cognitive load for speech comprehension (Cui and Herrmann, 2023; Mahanama et al., 2022; Šabić et al., 2020). However, our participants did not show this reduction in eye movement, highlighting the limitations of generalizing findings from passive listening tasks to real-life conversations. In face-to-face interactions, prolonged gaze on an interlocutor may violate social norms, and the additional demands of managing turn-taking and task-solving may contribute to more dynamic gaze behavior. This interpretation is supported by Hendrikse et al. (2022), who found increased eye movement in hearing-impaired listeners during virtual conversations. Pupil dilation, a well-established measure of cognitive load and listening effort (Mahanama et al., 2022; Naylor et al., 2018), showed a small but significant increase during communication in noise, consistent with findings by Aliakbaryhosseinabadi et al. (2023). However, the absence of differences in pupil dilation between listening and talking periods suggests that pupil dilation may reflect more than just effort-related mechanisms. It could also be influenced by general arousal or stress responses to the noisy environment, as pupil size is sensitive to a range of factors, including emotional arousal and environmental stressors (Mahanama et al., 2022). These results indicate that while measures such as eye movement and pupil dilation are valuable for assessing cognitive load and listening effort in controlled settings, they may not fully capture the challenges experienced during active participation in face-to-face conversations.

### Effect of conversational status

During listening, participants primarily directed their gaze toward their interlocutors, particularly the active talker. In contrast, while talking, they shifted their gaze between interlocutors and task materials, consistent with previous findings (Degutyte and Astell, 2021; Lu et al., 2021; Maran et al., 2021) Fixating on the current talker likely supported speech comprehension (Schwartz et al., 2004) and signaled attentiveness. In group conversations, the talker must monitor listeners for signs of attention and potential backchannels while simultaneously preparing their next utterance. Given that our task materials contained information relevant to speech preparation, it remains unclear whether talkers used these materials as a visual aid for turn preparation or intentionally averted their gaze from interlocutors - a behavior previously linked to cognitive demands (Degutyte and Astell, 2021; Ho et al., 2015).

We further analyzed the detailed dynamics of gaze behavior at the onset and offset of conversational turns. At the start of a turn, participants increased their gaze toward the current talker, while the talker often shifted their gaze toward the task material and maintained focus there after initiating their turn. This behavior may serve as a turn-holding strategy (Degutyte and Astell, 2021). Similar findings were reported by Ho et al. (2015), who observed this gaze pattern in dyad interactions involving clearly defined roles. Unlike Ho′s study, however, our conversations lacked explicit roles, and the triadic nature of the interactions required additional negotiation for turn-taking. Despite these differences, we observed a comparable gaze strategy where the current talker signals the next. While gaze avoidance at the onset of a turn may reflect cognitive load related to turn preparation, we anticipated greater gaze avoidance in noisy environments. However, in noisy conditions, participants increased their gaze toward interlocutors at the start of a turn, possibly reflecting a heightened need to support and monitor turn-taking through gaze behavior in these challenging settings.

At the end of a turn, gaze behavior becomes predictive of who will take the next turn. Listeners who do not intend to take the next turn tend to shift their gaze away from the current talker toward the upcoming talker or the task material. Conversely, listeners, who are preparing to speak, direct their gaze toward the task material earlier, likely to formulate their response and reduce eye contact with interlocutors. In general, a listener who intends to remain in the listening role signals their unwillingness to take the next turn by averting their gaze from the current talker and looking at the person who will become the next talker (Degutyte and Astell, 2021). The current talker, in turn, reduces their gaze on the task material and directs more attention toward the listener who will take over. These findings suggest that gaze serves as a critical nonverbal cue for negotiating turn-taking during conversation, helping participants coordinate their roles effectively in both quiet and noisy environments.

### Limitations and future work

We chose to have participants converse over a task to provide some control over the conversation’s content and increase the likelihood of a sustained engagement among all participants. Although the observed utterances were relatively long and participants did not have explicitly assigned roles – unlike other studies that used task-based conversations to examine turn-taking dynamics and conversational challenges (Aliakbaryhosseinabadi et al., 2023; Ho et al., 2015; Sørensen et al., 2021) - the constrained content may still have influenced vocal and movement behavior, potentially limiting the generalizability of our findings to more spontaneous, free-form conversations. Moreover, task-specific content may have shaped participants’ behavior depending on their prior knowledge of the topic, introducing variability distinct from that seen in unstructured dialogue. The inclusion of task material also introduced an additional visual area of interest, which may further affect generalizability to real-life conversations. However, as noted by Hendrikse et al. (2019, 2022), it is common for individuals to perform additional tasks while conversing, such as eating or taking notes in meetings. Stacey et al. (2020) also suggested that preferences for visual or auditory information vary depending on context and the information available. Future work could investigate how conversational behavior adapts in more realistic activities and how scene information influences these dynamics.

We used two distinct noise levels to elicit clear behavioral differences while ensuring that participants could sustain conversations without excessive fatigue. However, a recent study by Miles et al. (2023) suggests that our noisy condition may not have been sufficiently challenging, as certain behavioral changes only emerge at noise levels above 78 dB. Additionally, we employed multi-talker babble as background noise, which was likely unintelligible (Simpson and Cooke, 2005) and presented from a fixed direction, making it easier for participants to ignore. In real-world settings, noise directionality often varies, and background speech may sometimes carry relevant information for the listener. Aubanel et al. (2011) demonstrated that the presence of another group conversing in the same room significantly alters turn-taking dynamics, suggesting that nonverbal communication increases in such situations. It remains to be explored whether gaze behavior is similarly affected by more realistic and dynamic acoustic conditions.

Participants were seated in fixed positions during triadic conversations with unfamiliar confederates. Weisser et al. (2021) observed more pronounced behavioral adaptations, such as changes in conversational distance, when participants were standing rather than sitting, and when they were familiar with each other. This suggests that behavioral adaptations may vary depending on freedom of movement and familiarity among interlocutors. Future research could explore how conversational behavior evolves when participants have greater freedom of movement, including non-gaze behaviors such as facial expressions and gestures. Examining the interaction between various communication strategies and social norms in more naturalistic settings would provide a deeper understanding of the complex dynamics of human communication.

Our participant group was relatively homogeneous in terms of age, cultural background, and education, as all participants were young, native Danish speakers. These characteristics interact with cultural norms and may significantly influence gaze and turn-taking patterns (Degutyte and Astell, 2021). Additionally, age, conversational disabilities (e.g., hearing impairment), and cognitive abilities may influence how effectively individuals utilize visual information, potentially altering gaze behavior. While triads represent the minimal group size necessary to exhibit group dynamics distinct from dyadic conversations, the complexity of turn-taking negotiation increases with more interlocutors. Therefore, it is crucial to examine gaze behavior across a more diverse population and in a broader range of scenarios to assess the generalizability of these findings.

The absence of findings related to markers of cognitive load may stem from the low sampling rate of the eye tracker and the extent of head movement. Existing algorithms for segmenting gaze trajectories typically rely on fixed head positions and low noise in the eye-tracking data. While these measures perform well in controlled laboratory settings, real-world interactions often involve additional types of eye movements, such as smooth pursuit and vergence, which may convey valuable information about participants’ mental states. As wearable eye-tracking technology becomes more advanced, novel methods for labeling and analyzing these inherently noisier data will likely emerge, providing deeper insights into the functions of these eye movements. Measuring pupil dilation during conversation presents additional challenges, as eye movements and luminance variations can significantly influence pupil size, potentially obscuring effort-related variations. Although we attempted to mitigate these factors by regressing eye movement and luminance, their influence may persist over longer timescales. Establishing a reliable baseline for normalizing pupil data is further complicated by significant shifts in gaze position. Additionally, we did not assess participants’ stress levels, which may have influenced pupil responses (Mahanama et al., 2022). Future studies exploring pupillometry as a marker of communication difficulty may benefit from more stable gaze conditions, the inclusion of stress measures, or the use of advanced modeling techniques that better account for position and luminance variability.

The movement data collected in our study revealed significant differences between the two noise conditions and between listening and talking periods. As noise levels increased, gaze became more closely aligned with the focus of auditory attention and appeared more predictive of turn changes. This finding could inform the development of future communication aids designed to rapidly identify or even anticipate the focus of auditory attention and adapt signal processing - such as beamforming - accordingly (Favre-Félix et al., 2018; Kidd, 2017). However, the observation that participants directed their gaze toward the current talker only about half of the time highlights the need for more sophisticated algorithms to use gaze as a reliable estimator of auditory attention. Such algorithms would need to account for variability in gaze position and behavior influenced by the acoustic scene.

## Conclusion

This study investigated the effects of acoustic challenges on gaze behavior during triadic face-to-face conversations using wearable eye trackers. The results demonstrated that increased noise levels significantly influenced participants’ gaze patterns, leading to a greater focus on the current talker and enhancing the predictive power of gaze for turn-taking dynamics. Specifically, gaze was more stable and concentrated on the active talker during listening phases, while participants frequently shifted their gaze between interlocutors and task materials during speaking phases. These findings indicate that gaze behavior is sensitive to communication difficulties, with individuals relying more on visual cues when acoustic information is compromised. The increased alignment of visual and auditory attention in noisy conditions underscores the potential of incorporating gaze data to guide hearing aids visually. The realistic experimental setup provided high ecological validity, and the fine temporal resolution of our analysis at turn onset and offset offered valuable insights into the interaction between gaze and turn-taking behavior. Overall, our findings suggest that gaze serves as an ecologically valid marker of communication difficulties, with implications for the design of future hearing aids. Such devices could leverage gaze data to assess the acoustic scene and monitor the user’s attention, ultimately improving user experience in challenging communication environments.

## Data Availability

The data used for the speech material creating the background noise used in this study is available here: https://data.dtu.dk/articles/Recordings_of_Danish_Monologues_for_Hearing_Research/9746285. The segmented speech and movement data of this study is available here: https://doi.org/10.11583/DTU.29666606. Further inquiries can be directed to the corresponding author.
